# *Isocitrate dehydrogenase 2* regulates the proliferation of triple-negative breast cancer through the ferroptosis pathway

**DOI:** 10.1038/s41598-024-55561-0

**Published:** 2024-02-27

**Authors:** Chengwu Zhang, Yuanhong Zhou, Tao Chen, Sudhanshu Bhushan, Shengrong Sun, Panshi Zhang, Yalong Yang

**Affiliations:** 1 Department of Thyroid and Breast Surgery, Hubei General Hospital, Renmin Hospital of Wuhan University, Wuhan, 430000 China; 2 Department of Thyroid and Breast Surgery, Yichang Central People’s Hospital, The First Clinical College of Three Gorges University, Yichang, 443000 China; 3 The First Clinical College of Three Gorges University, Yichang, 443000 China; 4 Yichang Central People’s Hospital, The First Clinical College of Three Gorges University, Yichang, 443000 China; 5 Department of Anatomy and Cell Biology, Unit of Reproductive Biology, Justus-Liebig-University Giessen, 35390 Giessen, Germany; 6 Department of Thyroid and Breast Surgery, Tongji Hospital, Huazhong University of Science and Technology, Wuhan, 430000 China; 7 Department of Breast Surgery, Hubei Cancer Hospital, Huazhong University of Science and Technology, Wuhan, 430000 China

**Keywords:** Ferroptosis, Triple-negative breast cancer, Proliferation, IDH2, Breast cancer, Cancer genetics, Cancer metabolism

## Abstract

Triple-negative breast cancer (TNBC) is currently the type of breast cancer with the worst prognosis; it lacks specific treatments, such as ER/PR antagonistic endocrine and anti-HER2 targeted therapies. Although immunotherapy with immune checkpoints has shown some efficacy in many solid tumors, clinical data in TNBC suggest significant limitations. The essence of ferroptosis is the impaired metabolism of intracellular lipid oxides, which in turn causes the activation and abnormalities of the immune system, including ROS, and not only plays an important role in liver injury and organ aging but also a large amount of data points to the close correlation between the ferroptosis process and tumor development. In this study, through the analysis of large-throughput biological data of breast tumors, combined with the characteristics of the biological process of ferroptosis, the specific gene *IDH2* was found to be significantly highly expressed in TNBC and functionally correlated with ferroptosis. Through clinical specimens validated at the gene and protein levels, in vitro tumor cell line validation, and in vivo mouse models, we found that the high expression of *IDH2* in TNBC has a role in inhibiting the ferroptosis process in TNBC, thus promoting the proliferation of TNBC cells and other malignant features.

## Introduction

Breast cancer (BRCA) has surpassed lung cancer as the most prevalent malignancy in humans^1^. Although molecular staging further divides breast cancer into ER (estrogen receptor)-positive and/or PR (progesterone receptor)-positive luminal types, Her2 (human epidermal growth factor receptor 2)-positive, and triple-negative breast cancer (TNBC) types, as well as concomitant endocrine and targeted therapies, it is still difficult to improve therapeutic outcomes for some types of breast cancer, such as endocrine-resistant breast cancer, inflammatory breast cancers, and TNBC^2–4^. Although immunotherapy with immune checkpoints (such as PD-1 (programmed cell death protein 1) and PDL-1 (PD-1 ligand 1) antibodies) has shown some efficacy in many solid tumors, including breast cancer, clinical data in TNBC suggest significant limitations^5–7^. Ferroptosis was presented in 2012 as a novel but “ancient” mode of regulated programmed cell death, which is mainly characterized by disturbed mitochondrial metabolism and intracellular phospholipid peroxidation^8–10^. With further research, the classical GPX4 (glutathione peroxidase 4)-dependent SLC7A11-GSH-GPX4 axis and GPX4-independent NAD(P)H-FSP1-CoQ10 and DHFR-BH4-GCH1 axis regulatory mechanisms have been identified^11–15^. Accumulating research suggests that the occurrence and regulatory mechanisms of many human diseases and tumor models are closely related to the ferroptosis process, and intervention in the ferroptosis process has become an effective measure for the treatment of many diseases and tumors^9,10,16–19^. *IDH2* (isocitrate dehydrogenase 2) is one of the most critical genes for bioenergy metabolism, playing an essential role in the redox process of NAD(P)/NADPH and a key regulatory role in the cellular ROS (reactive oxygen species) oxidative stress response^20–22^. Although many studies have found that mutations in the *IDH2* gene are commonly found in hematological tumors and gliomas, the mechanism is the gain-of-function effect of the mutation and the DNA damage mechanism of the abnormal accumulation of the metabolite D2-HG (D-2-hydroxyglutaric acid)^23–25^. However, gradually, researchers have found that the expression level of wild-type *IDH2* has a certain correlation with the occurrence and development of some human diseases, including carcinoma^26–28^.

Therefore, by analyzing the data through bioinformatics, this study will, for the first time, investigate the mechanism of the correlation between ferroptosis sensitivity and abnormally high expression of *IDH2* in TNBC cancer cells in their highly proliferative biologic characteristics, which will provide new ideas and potential targets for the treatment of TNBC.

## Methods

### Animals

Adult BALB/c Nude female mice (6–8 weeks, 22 ± 1.5 g) were purchased from Gempharmatech Co., Ltd. (Jiangsu, China) and kept under standard conditions (22 °C, 12 h light/dark cycle) with pelleted food and water ad libitum. The study was carried out under a project license (No.: 20200702) granted by the Laboratory Animal Welfare and Ethics Committee, Renmin Hospital of Wuhan University, in compliance with Institutional Animal Care and Use Committee guidelines for the care and use of animals, and all process were in compliance with ARRIVE guideline. Animals were sacrificed with appropriate way and anaesthetised with isoflurane followed by cervical dislocation.

### Cell culture and lentiviral transfection

MCF-10A, MB-231, MCF-7 and AU-565 were purchased from ATCC (American Type Culture Collection) with STR (Short Tandem Repeat) certifications and were cultured in DMEM (Gibco, USA) with 10% FBS (Cellbox, China) and 1% P/S (Procell, China) at 37 °C and 5% CO_2_ conditions, except for MCF-10A, which was cultured in special medium (CM-0525, Procell, China).

MB-231 cells were cultured in 6-well plates at 2 × 10^5^ cells per well, and 12 h later, virus infection (pSLenti-U6-shRNA(IDH2)-CMV-EGFP-Puro-WPRE, 50 nmol/L) and 10 μL of 1 mg/mL polybrene were added to each well, making a final working concentration of 5 μg/mL. The medium was changed after 12 h of infection. After 72 h, a final concentration of 1 μg/mL puromycin was added. Fresh medium was then changed every 2–3 days with a final concentration of 1 μg/mL puromycin. After 4 weeks of drug screening, fluorescence photographic screening was performed. Three different siRNA-IDH2 (NM_002168.4) were constructed and screened the one with the best knockdown effect for subsequent experiments. The target sequence of the siRNA was CCAAGAACACCATACTGAAAG.

### Proliferation analysis: EdU assay and colony formation

MB-231 cells were transfected into 6-well plates with siRNA-*IDH2* or si-negative control (si-NC). After 24 h, the cells were washed with DPBS and counted. Subsequently, we plated cells at a low density (200 cells per well) into 6-well plates to form clones. After 2 weeks, we used crystal violet (Solarbio, China) to stain the colonies, and the number of clones in each plate was counted.

EdU assay was carried out according to the manuscript from APExBIO (K1077). siRNA-*IDH2*-MB231 or si-NC-MB231 cells were seeded at 2 × 10^5^ cells/well into 6-well plates with glass, and 10 mM EdU was added to an equal volume of cell culture medium to a working concentration of 10 µM/well and incubated at 37 °C for 2–4 h. Cells were then washed, fixed and permeabilized using DPBS, 4% PFA and 0.5% Triton X-100 sequentially. Then, click buffer was added to the cells, and the nuclei were stained with DAPI. Cells were screened with Olympus IX73.

### Transwell and wound healing

siRNA-*IDH2*-MB231 or si-NC-MB231 cells were seeded at 2 × 10^5^ cells/well into 6-well plates at 37 °C. When the cells were 90% confluent, the cells were scraped off vertically into a straight line utilizing a sterile 200 µL tip. The floating cells were gently washed off, supplemented with fresh serum-free medium, photographed at 0 h, 24 h and 48 h, and quantitatively analyzed using ImageJ.

siRNA-*IDH2*-MB231 or si-NC-MB231 cells were collected at 2 × 10^4^ cells/tube into sterile EP tubes. The cell suspension was blown and added into the Transwell chambers (Corning Incorporated). Then, 500 μL of complete medium containing 10% FBS was added to the Transwell plates, and the chambers were placed into the plates and incubated in a CO_2_ (5%) incubator at 37 °C for 24 h. The chambers were removed, the medium was washed off with PBS and stained with 0.1% crystal violet staining solution for 10 min, and the surface was washed with water to remove the crystal violet. The cells in the upper chamber were wiped out with a cotton swab, and the noncellular inoculated side was photographed under an inverted microscope.

### Immunohistochemistry (IHC)

Duplicate paraffin sections were deparaffinized, and IHC staining for IDH2 was performed using rabbit anti-IDH2 polyclonal antibody (pAb) (1:100, ABclonal, China) and HRP goat anti-rabbit IgG (H + L) (AS014, ABclonal, China). IHC quantification was performed using ImageJ software.

### Western blotting (WB)

siRNA-*IDH2*-MB231 or si-NC-MB231 cells were collected from fully grown 6-well plates for total protein using a ProteoPrep Kit (NA.32, Merck) and measured by a BCA Protein Assay Kit (P0011, Beyotime, China). Forty micrograms of protein from each sample was added for SDS-PAGE. Proteins were then transferred to 0.45 µm PVDF Transfer Membranes (Millipore) at 100 V for 1 h. The membrane was then blocked with 5% BSA and incubated with the indicated primary antibodies against β-actin, IDH2, PI3K/p-PI3K, AKT/p-AKT and mTOR/p-mTOR (ABclonal, China) overnight at 4 °C. Then, the membrane was washed and incubated with HRP goat anti-rabbit IgG (H + L) (AS014, ABclonal, China). Images were screened by Tanon 5200 (China) using an Ultra High Sensitivity ECL Kit (HY-K1005, MCE). Figures were cropped for better demonstration.

### Total RNA extraction and qPCR analysis

RNeasy plus mini kits (Qiagen) were used to extract total RNA according to the protocol provided by the manufacturer. qRT-PCR was conducted in triplicate with 2xSYBR Master Mix (ABclonal). *GAPDH* was used as an internal control, and the 2^−ΔΔCt^ values were normalized to relative levels. The primer sequences for qPCR used in this study are shown in Supplementary Table 1.

### Detection of the cell cycle and apoptosis using flow cytometry (FC)

siRNA-*IDH2*-MB231 or si-NC-MB231 cells were collected from 90% fully grown 6-well plates into FC tubes. Cells were washed with cold PBS and resuspended in 500 µL of 1 × binding buffer (Annexin V-APC/7-AAD apoptosis kit, Multi Sciences, China) and 5 μL of Annexin V-APC, followed by 10 μL of 7-AAD. The apoptosis assay was measured in 5 min on a BD FACSCalibur.

Cells were prepared as described above and washed with cold PBS. Then, the cells were fixed with 500 µL of 75% ethanol at 4 °C overnight. PI/RNase A was prepared as indicated (C1052, Beyotime, China) and incubated with cells for 30 min in the dark. The samples were analyzed on a BD FACSCalibur and FlowJo 10.

### Mouse tumor model

A total of 2 × 10^6^ siRNA-*IDH2*-MB231 or si-NC-MB231 cells were collected in PBS and injected into the subcutaneous interstitial space of the right forelimb of the mice. The mice were checked every day, and the length and width of the tumor were recorded when the injected subcutaneous tissue developed a bulge visible to the naked eye on Day 7. After that, mice were intraperitoneally injected with erastin (20 mg/kg) or PBS as a control every day.

### In vivo imaging of mice

The transfected virus has GFP (green fluorescent protein) suitable for in vivo imaging. Seven days after the subcutaneous loading of the mice, the tumors in the NC group were significantly enlarged to approximately 7 cm, and we performed intraperitoneal injections of erastin and decided to terminate the observation after 7 days based on the fact that the tumors in the mice with the smallest tumors were significantly reduced and close to disappearing, as observed by the naked eye. Mice were anesthetized with isoflurane inhalation and placed in the optimal position for imaging photographs (PerkinElmer IVIS Lumina III).

### Bioinformatics analysis

The breast cancer dataset (GSE21653) was acquired from The Gene Expression Omnibus (GEO, https://www.ncbi.nlm.nih.gov/geo/) database, based on GPL570 platforms ([HG-U133_Plus_2] Affymetrix Human Genome U133 Plus 2.0 Array) and including a total of 266 samples of breast cancer. Raw data quality control, data preprocessing, and weighted gene coexpression network analysis (WGCNA) were carried out by using R software (Version 4.0.3), as described in our primary paper^29^. Principal component analysis (PCA), functional enrichment analysis, protein‒protein interaction network (PPI), expression analysis, and survival analysis were performed using Sangerbox tools (http://www.sangerbox.com/tool), Metascape web (http://Metascape.org), and STRING online database (version 11, http://string-db.org/).

UALCAN web resource (http://ualcan.path.uab.edu/analysis-prot.html), and Breast Cancer Gene-Expression Miner v4.7, respectively. Cytoscape (version 3.9.1) software was used for PPI visualization.

### Ethical approval

The authors take responsibility for all aspects of this work to ensure the accuracy and completeness of the content. The patient samples used in this study were approved by Wuhan University People's Hospital (No.: 20133655893). All patients signed an informed consent form for the data collection. All methods were approved by the Research Ethics Committee of Wuhan University People's Hospital, and this study was conducted in accordance with declaration of Helsinki.

### Statistical analysis

All the data are shown as the mean ± SD. The results were analyzed using GraphPad Prism version 9 (GraphPad Software, Inc.), and significant differences between means were tested using one-way ANOVA followed by Tukey’s test. A significance level of p < 0.05 was used.

## Results

### TCGA data analysis and GO analysis to find DEGs in BRCA

**Figure 1:**
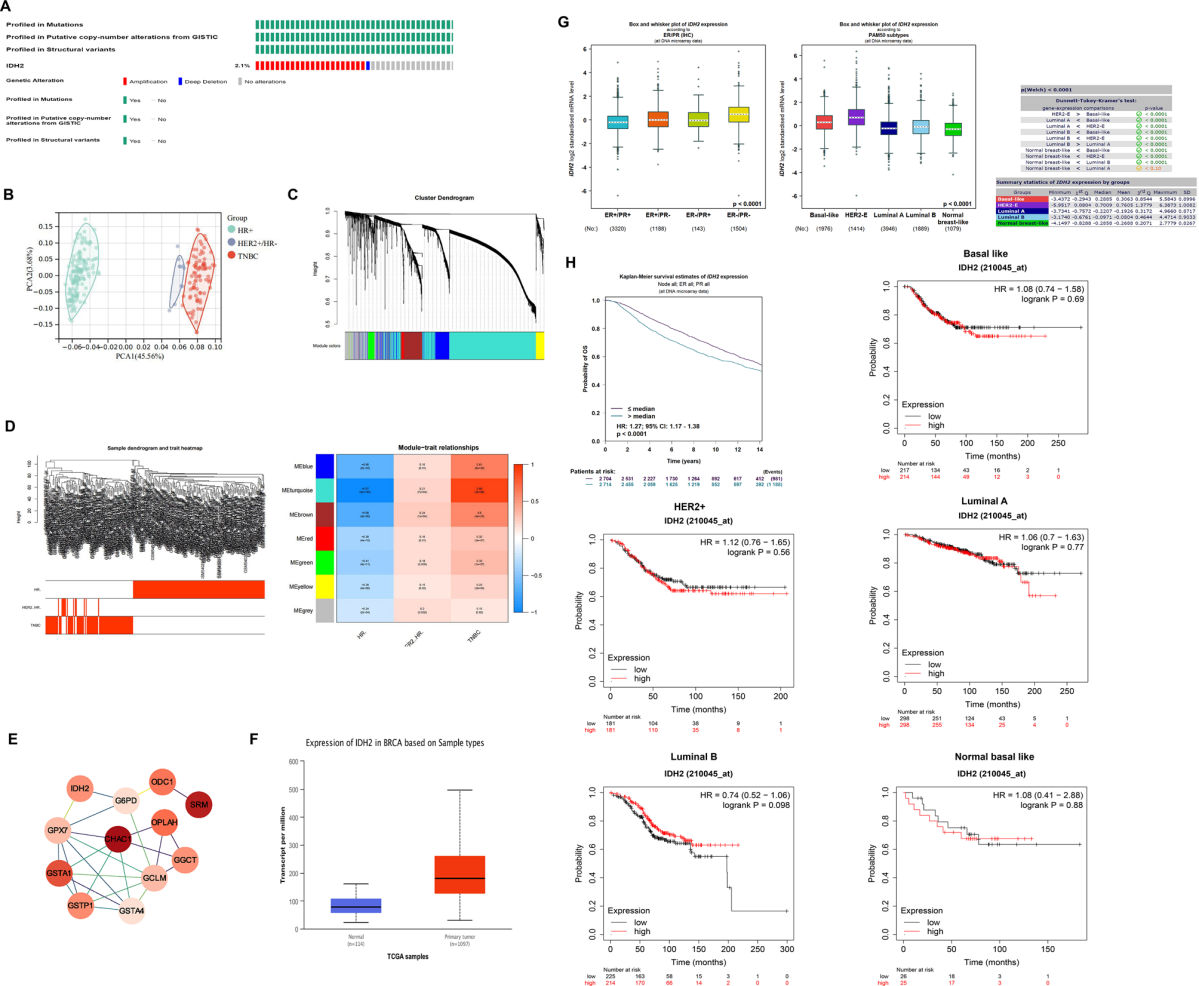
TNBC-specific module detection and ferroptosis-related gene *IDH2* identification. (**A**) The mutation profile of *IDH2* in breast cancer, showing only 2.1% mutation with amplification mode (https://www.cbioportal.org/). (**B**) PCA plot based on the RNA expression level of all samples used for WGCNA. (**C**) Hierarchical clustering dendrogram of the top 20% variant genes. The color of the row underneath the dendrogram displays the corresponding module assignment determined by the Dynamic Tree Cut. One color represents one module. (**D**) Heatmap of the correlations between module eigengenes and various subtypes of breast cancer samples. The table is color-coded by correlation based on the color legend; blue represents a negative correlation, while red represents a positive correlation. The number in the rectangle is the concrete correlation coefficient and p value (in the bracket). (**E**) PPI network constructed by hub genes that are enriched in the glutathione metabolism pathway. Each node represents a gene. The red color represents the positive significance to TNBC. The darker the nodes, the more significant the gene is to TNBC. (**F**) *IDH2* expression level between normal breast samples and breast cancer samples. (**G**) IHD2 protein levels among four molecular subtypes of BRCA, including PMA50 subtypes. (**H**) The probability of OS analysis between *IDH2* expression levels in BRCA patient and each PMA50 subtype.

First, we analyzed the mutation rate of *IDH2* in BRCA, and found only 2.1% of mutations occurred with mostly amplification type (Fig. 1A). Thus we mainly focused on the expression status of *IDH2* in BRCA, and we used the single-cell sequencing data of breast cancer from the TCGA (The Cancer Genome Atlas) public database for clustering analysis of differentially expressed genes (DEGs) between different molecular subtypes of BRCA and found that *IDH2* was significantly highly expressed in TNBC through GO functional enrichment analysis and ferroptosis pathway aggregation analysis, and the high expression status of *IDH2* showed a significant positive correlation with poor prognosis of BRCA (Fig. 1B–H). Meanwhile, data also indicated that possibilities of OS showed even different patterns with *IDH2* expression status under different PAM50 subtypes (Fig. 1H).

### TNBC has a relatively higher expression of IDH2 at both the gene and protein levels than other molecular types

**Figure 2:**
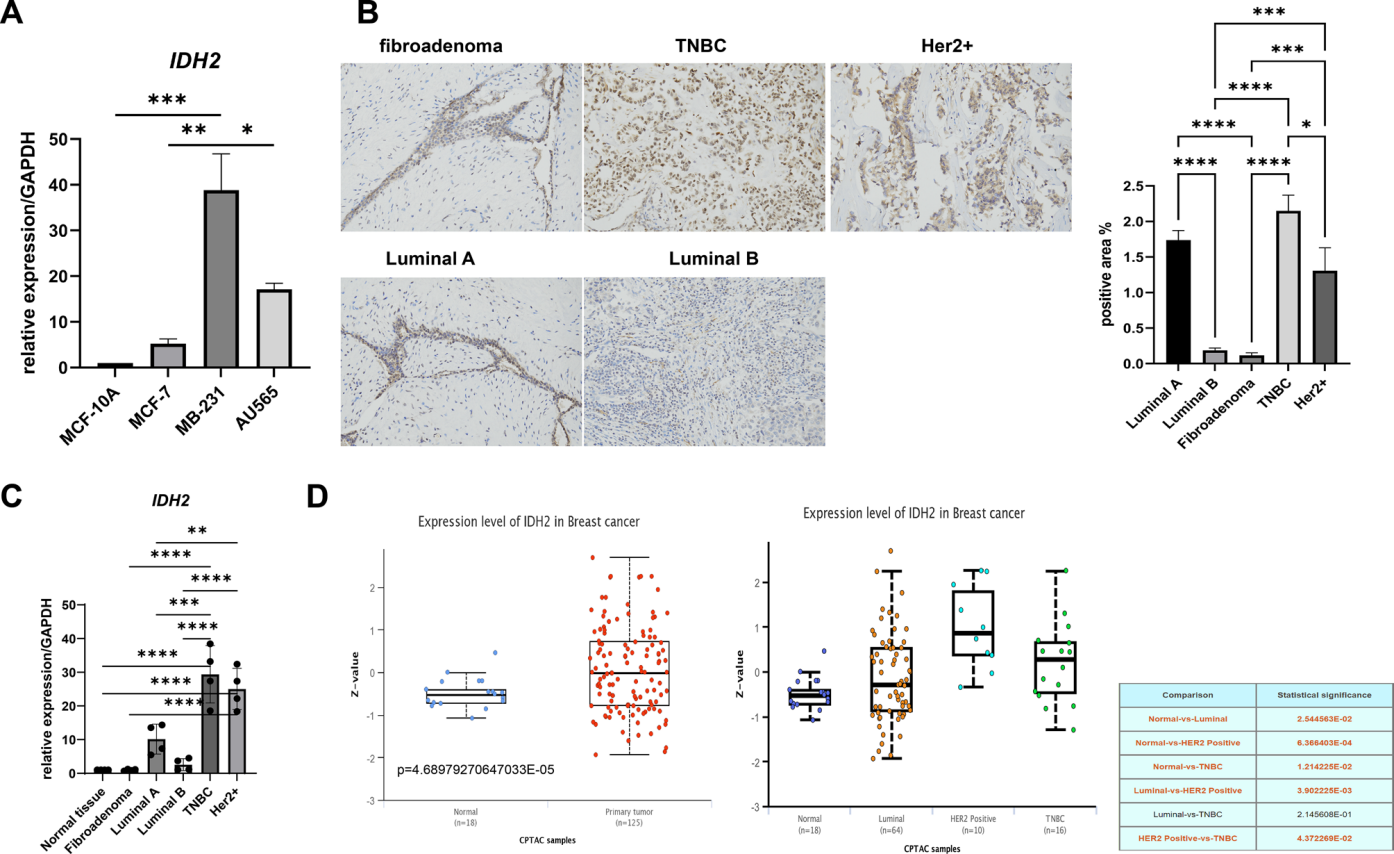
TNBC showed a significantly higher expression level of IDH2 than other molecular types. (**A**) The expression level of *IDH2* was measured by qRT‒PCR among different molecular types, and CT values were normalized to GAPDH as a housekeeping gene. n = 4. (**B**) IHC staining was carried out to measure the expression of IDH2 among fibroadenoma, luminal A, luminal B, Her2+ and TNBC tissue samples. Pictures shown at 200× magnification. n = 4. (**C**) The expression level of *IDH2* was measured by qRT‒PCR among different molecular tissues, and CT values were normalized to GAPDH as a housekeeping gene. n = 4. (**D**) Expression profiles of IDH2 in different BRCA subtypes using UCLCN datasets. (https://ualcan.path.uab.edu/cgi-bin/CPTAC-Result.pl?genenam=IDH2&ctype=Breast) One-way ANOVA followed by Tukey’s test was used to analyze the statistical discrepancy. *p < 0.05, **p < 0.01, ***p < 0.005, ****p < 0.001.

To further verify the bioinformatics data shown above, we tested the gene expression level of *IDH2* among different breast cancer molecular typing cell lines in vitro, including MCF-10A (normal human mammary epithelial cells), MB-231 (TNBC subtype cells), MCF-7 (luminal subtype cells), and AU565 (Her2+ subtype cells). As the results showed, the gene expression of *IDH2* in MB-231 cells was significantly higher than that in MCF-10A, MCF-7 and AU565 cells (Fig. 2A). Furthermore, we analyzed the protein levels of IDH2 among fibroadenoma, luminal A, luminal B, Her2+ and TNBC tissue samples. The data indicated that the protein level of IDH2 in TNBC was much higher than that in Her2+, luminal B and fibroadenoma, and importantly, benign fibroadenoma had the lowest level of IDH2 (Fig. 2B). Further, we used Clinical Proteomic Tumor Analysis Consortium and the International Cancer Proteogenome Consortium datasets to further explore IDH2 protein expression levels among different subtypes of breast cancer (Fig. 2D). Figure 2C suggests that the *IDH2* expression level of breast cancer is significantly higher than that of normal breast tissue, while TNBC and HER2+ subtypes are significantly higher than Luminal subtype. In summary, the above data showed that breast cancer generally had higher levels of IDH2 at both the gene and protein levels than benign tissue, and most importantly, TNBC had higher expression level of IDH2 compared with other molecular types.

### Knocking down the expression level of *IDH2* in MDA-MB-231 cells can inhibit the proliferation and invasive ability of TNBC in vitro

**Figure 3:**
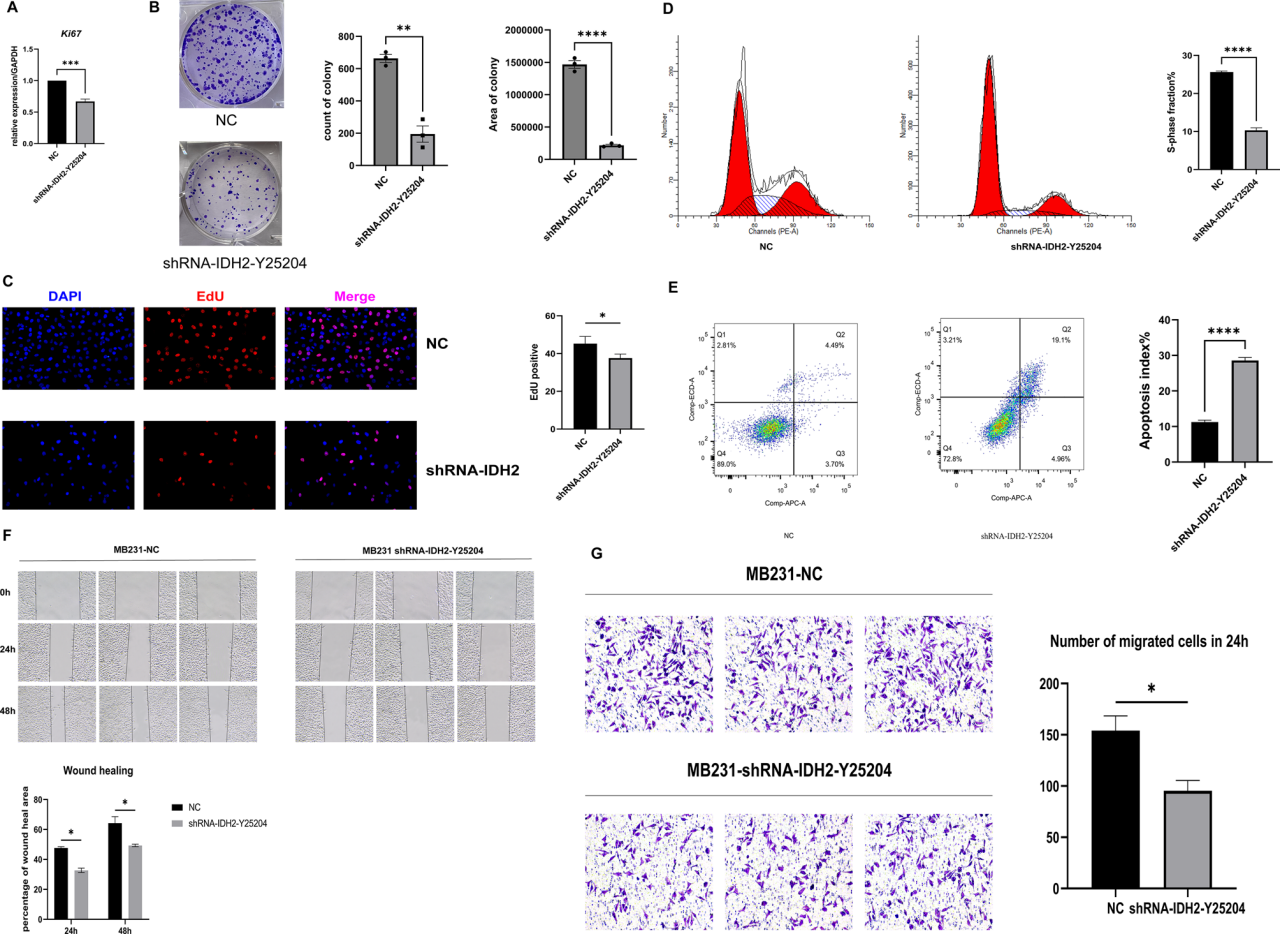
Reduced cell proliferation and migration after *IDH2* knockdown in MB-231 cells. (**A**) The expression level of *Ki-67* was measured by qRT‒PCR between MB-231-sh-NC and MB-231-shRNA-*IDH2* cells. n = 4. (**B**) A colony formation assay was carried out with MB-231-sh-NC and MB-231-shRNA-*IDH2* cells. The count and area of the colony were measured, and a *t* test was used. n = 3. (**C**) The cell cycle was measured by FC between MB-231-sh-NC and MB-231-shRNA-*IDH2* cells. The S phase percentage was compared, and a *t* test was used. n = 3. (**D**) EdU assay was carried out using the EdU Kit, and pictures were analyzed by ImageJ. A *t* test was used. n = 3. (**E**) Apoptosis of cells was measured by FC and analyzed using a *t* test. n = 3. (**F**) A wound healing assay was carried out as mentioned in the methods section, and pictures were taken at 0 h, 24 h and 48 h. The results were analyzed by ImageJ and compared using a *t* test. n = 3. (**G**) Transwell assays were carried out as described above, and pictures were taken at 24 h. n = 3. Cell numbers were analyzed by ImageJ and compared using a *t* test. n = 3. *p < 0.05, **p < 0.01, ***p < 0.005, ****p < 0.001.

From the above data, we can see that the TNBC subtype with high proliferative capacity and malignancy had the highest IDH2 gene and protein expression levels, so we further verified whether the high *IDH2* expression level was positively correlated with the proliferative capacity of TNBC. We first constructed a lentiviral-packaged sh-*IDH2* plasmid, screened the stable and most effective knockdown plasmid, shRNA-*IDH2*-Y25204 (SFig. 1A–C), and found a significant reduction in the expression of *Ki67* at the gene level after stable transfection to knock down the expression level of *IDH2* in the MB-231 cell line (Fig. 3A). Meanwhile, a plate cloning assay suggested that shRNA-*IDH2* significantly inhibited the proliferation ability of a single cell clone of MB-231 (Fig. 3B), and EdU fluorescence staining suggested that the proliferation division ratio of MB-231-shRNA-*IDH2* was significantly reduced compared with that of the NC group (Fig. 3C). Meanwhile, we examined the cell cycle and apoptosis of MDA-MB-231 cells after knockdown of *IDH2*. MB-231-shRNA-*IDH2* S-phase was significantly reduced (Fig. 3D), indicating a reduced proliferation ability, and the proportion of apoptotic cells was significantly increased (Fig. 3E). The plate scratch healing assay further suggested that the migratory ability of MB-231-shRNA-*IDH2* cells was significantly reduced at both 24 h and 48 h (Fig. 3F). Transwell assays also demonstrated that MB-231-shRNA-*IDH2* significantly inhibited the invasive ability of MB-231 cells (Fig. 3G). The above in vitro cellular assay data as a whole indicated that the high expression of *IDH2* in MB-231 cells can promote their proliferative capacity and malignant invasive ability.

### *IDH2* regulates the ferroptosis process in TNBC

**Figure 4:**
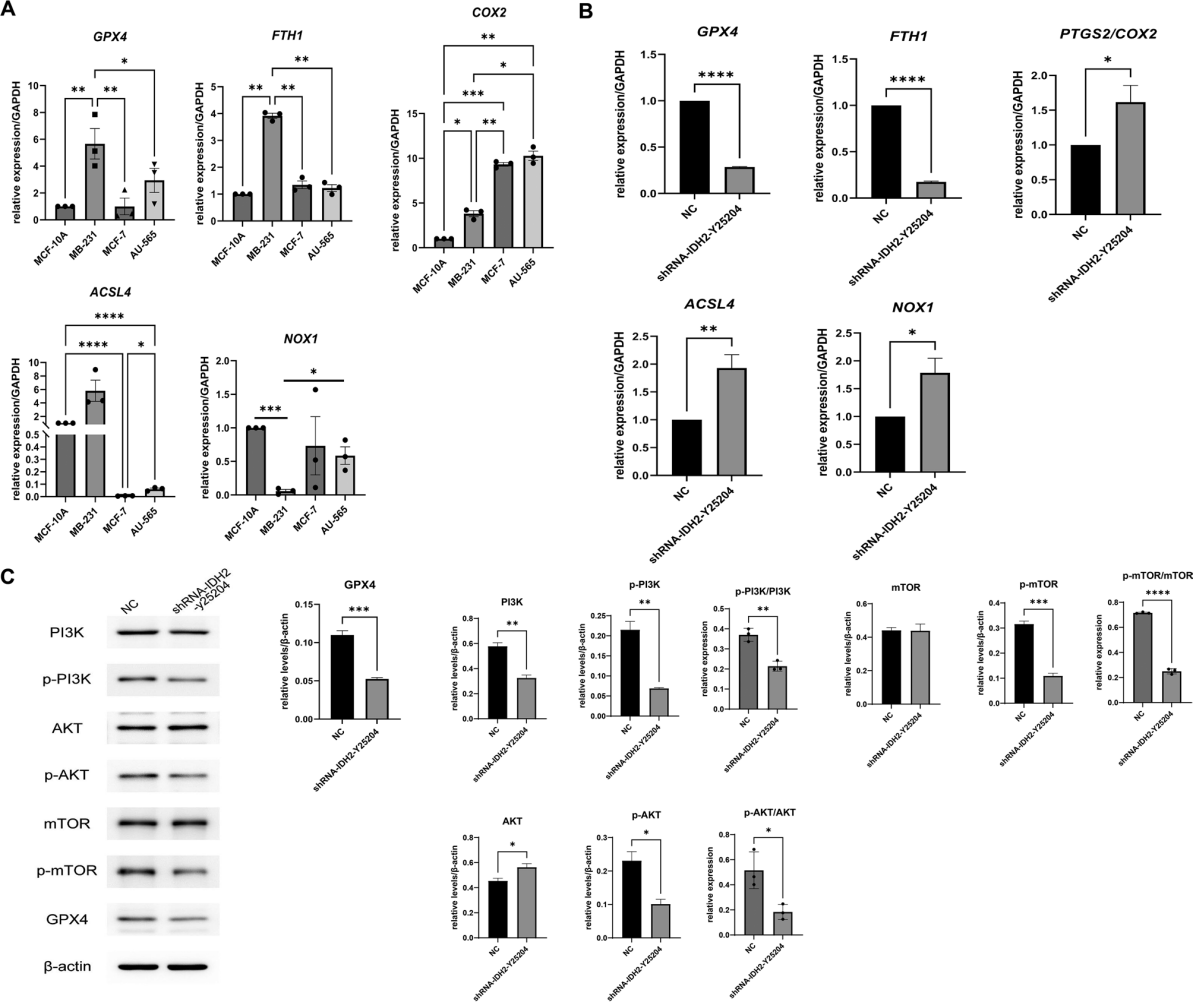
*IDH2* knockdown in MB-231 cells affects the expression of ferroptosis-related genes. (**A**) Expression levels of *GPX4, FTH1, COX2, ACSL1* and *NOX1* were measured by qRT‒PCR among different molecular types. n = 4. (**B**) Expression levels of *GPX4, FTH1, COX2, ACSL1* and *NOX1* were measured by qRT‒PCR between MB-231-sh-NC and MB-231-shRNA-*IDH2* cells. n = 4. (**C**) Protein levels of GPX4, PI3K/p-PI3K, AKT/p-AKT and mTOR/p-mTOR were measured by WB and compared with β-actin. Data were analyzed by one-way ANOVA followed by Tukey’s test and *t* test. n = 4. *p < 0.05, **p < 0.01, ***p < 0.005, ****p < 0.001.

Since the expression level of *IDH2* significantly regulated the proliferative capacity of TNBC cell lines and the most important process of *IDH2* in the physiological process is related to oxidative stress^31^, the expression level of *IDH2* was closely related to the ferroptosis process based on GO functional analysis. We first analyzed the expression status of *GPX4*, a classical mechanism of the ferroptosis pathway in BRCA, especially in TNBC, using public databases (SFig. 2). *GPX4*, a critical ferroptosis inhibitory system, was significantly expressed at low levels in BRCA and had the lowest expression level in TNBC among the four molecular subtypes (SFig. 2A), which suggested that BRCA and especially TNBC have a significantly high ferroptosis sensitivity. Additionally, another ferroptosis inhibitory gene, *FTH1,* was highly expressed in BRCA and showed no obvious difference among different subtypes (SFig. 2B). Of the other three pro-ferroptosis genes, *ACSL4* and *PTGS2* were downregulated in BRCA, while *NOX1* was highly expressed in BRCA (SFig. 2C). To our surprise, the bioinformatics data suggested that BRCA has higher levels of ferroptosis than normal breast tissue. This may be due to the relative deficiency of energy metabolism and oxygen metabolism in breast cancer tissues with overgrowth of cancer cells, causing low expression of the compensatory GPX4 inhibitory system as well as high levels of ferroptosis. Therefore, in the next step, we further validated the above data by examining the expression levels of ferroptosis-related genes, including the ferroptosis inhibitory genes *GPX4* and *FTH1* and the ferroptosis promoter genes *ACSL4*, *NOX1*, and *COX2*, using different BRCA cell lines. We found that at the cell line level, the expression levels of *GPX4* and *FTH1* were the highest in MB-231 cells. At the same time, genes related to the promotion of ferroptosis, including *ACSL4, NOX1* and *COX2*, showed the expected consistently low expression levels in MB-231 cells (Fig. 4A). Therefore, we next explored whether knockdown of *IDH2* expression levels altered the extent of ferroptosis in TNBC. qRT‒PCR results suggested that the expression levels of *ACSL4*, *NOX1* and *COX2*, which promote the ferroptosis process, were significantly increased, whereas the expression levels of *GPX4* and *FTH1*, which inhibit the ferroptosis process, were significantly decreased, and the changes in gene levels suggested that the ferroptosis activity or sensitivity of MB-231-shRNA-*IDH2* cells was increased (Fig. 4B). Next, we verified the levels of ferroptosis- and cell proliferation-related proteins, including GPX4, PI3K/p-PI3K, mTOR/p-mTOR and AKT/p-AKT. WB results again confirmed the reduced level of the ferroptosis-related protein GPX4 in MB-231-shRNA-*IDH2* cells (Fig. 4C), as well as the PI3K/AKT/mTOR signaling pathway. The data indicated that p-PI3K/PI3K, p-AKT/AKT and p-mTOR/mTOR reduced significantly in siRNA-*IDH2*-MB231 than siRNA-NC, which indicated reduced cell proliferation. In summary, our data showed that TNBC cells had significantly reduced ferroptosis sensitivity, while MB-231 cells with *IDH2* knockdown showed significantly increased ferroptosis sensitivity at both the ferroptosis-related gene and protein levels, as well as significant inhibition of tumor cell proliferation and other related malignant indicators.

### MB231 cells with low *IDH2* expression have reduced tumorigenicity in vivo

**Figure 5:**
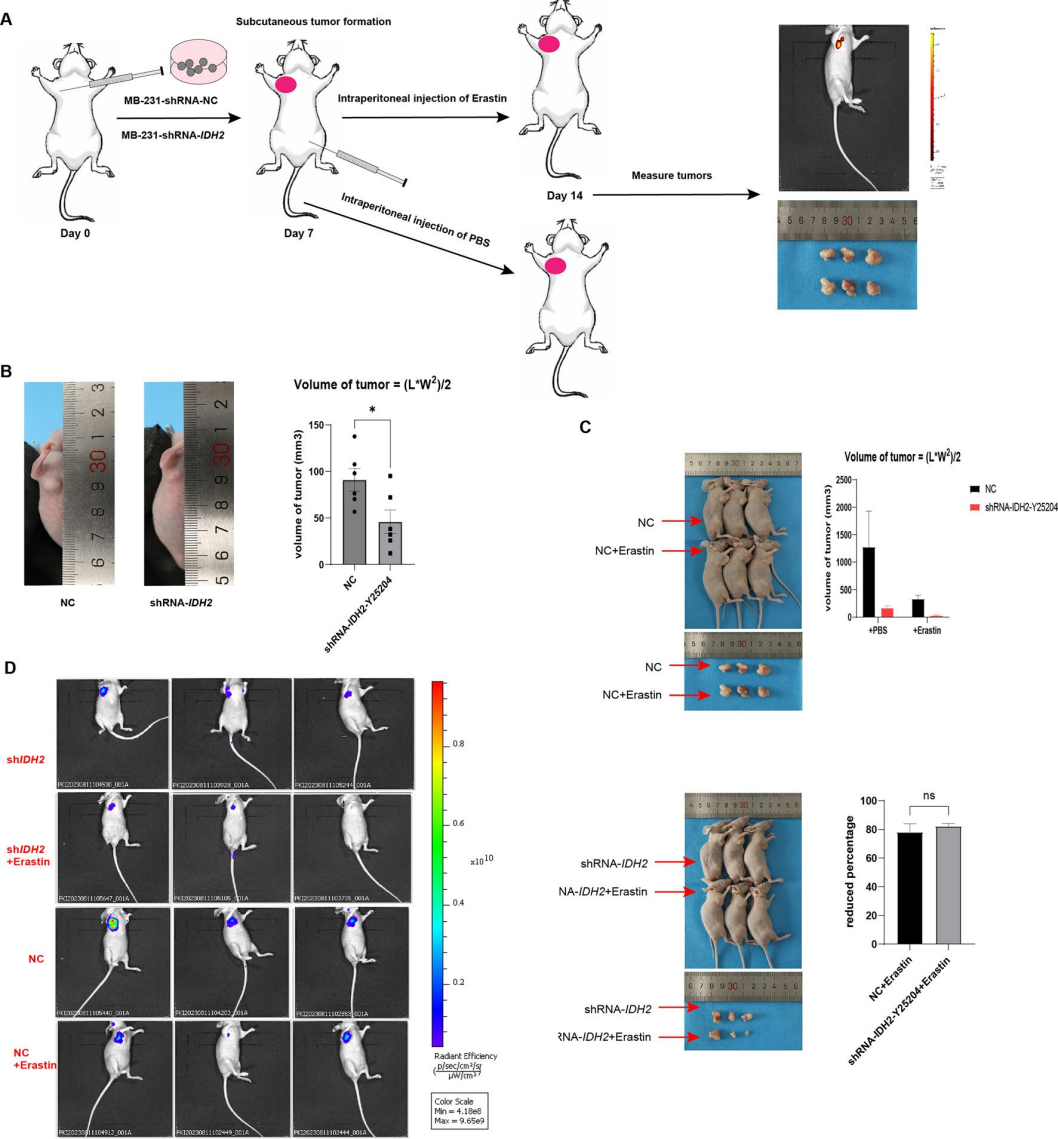
*IDH2* knockdown in MB-231 cells inhibits the extent of subcutaneous tumorigenesis in mice. (**A**) The working flowchart of the in vivo mouse model. (**B**) On Day 7 after subcutaneous injection of tumor cells, the sizes of tumors in the NC and shRNA-IDH2 groups were measured. Tumor volume = (L(length) * W^2^ (width))/2. Data were analyzed by *t* test. n = 6. (**C**) From Day 7, three mice were randomly selected from each group and injected intraperitoneally with erastin as described above. On Day 14, tumors were measured in vivo*,* and (**D**) mice were measured by in vivo imaging. The volume of tumors was compared among the four groups, and the reduced percentage of tumors was compared between the erastin-treated and PBS-treated groups. Data were analyzed by *t* test. n = 3. *p < 0.05.

According to the above results, we verified at the ex vivo level that the TNBC cell line MB-231 has a high expression level of *IDH2* and a low sensitivity to the ferroptosis process and further verified that *IDH2* affected the malignant characteristics of MB-231, such as proliferative and migratory abilities, by modulating the degree of the ferroptosis process through *IDH2* knockdown interference experiments. Next, we performed in-depth validation of an in vivo subcutaneous tumorigenic model using immunodeficient mice and MB-231 cells with stable knockdown of *IDH2*. We also utilized intraperitoneal injection of erastin (a classical iron death inducer) in mice to compare the degree of ferroptosis induced by shRNA-*IDH2* with that of controls. We selected 12 immunodeficient mice with similar body weights at 6–8 weeks and divided them into NC control, shRNA-*IDH2*, NC + erastin, and shRNA-*IDH2* + erastin groups, with 3 mice in each group (see Fig. 5A flow chart). First, we found that mice inoculated with the same number of MB-231 tumor cells showed a significant volume of subcutaneous tumor tissue in the NC group at 7 days, whereas mice in the shRNA-*IDH2* group had significantly smaller subcutaneous tumors, which were approximately 60% of the subcutaneous tumors in the NC group (Fig. 5B). After that, we performed daily intraperitoneal injections of 20 mg/kg erastin to treat the mice. After drug treatment, the NC group showed a significant increase in tumor shrinkage, while the shRNA-*IDH2* group showed no significant change (NC + erastin vs. shRNA-*IDH2* + erastin = 83.63 ± 2.616 vs. 12.55 ± 2.116, p < 0.0001, Fig. 5B–D). The results of the in vivo tumorigenic model in mice further suggested that the high expression level of *IDH2* regulated the tumorigenic ability of MB-231 cells mainly by modulating the degree of ferroptosis.

In summary, by analyzing the gene expression profiles of single-cell sequencing of breast cancer tumors in large passages, as well as screening genomics for functional enrichment and genes related to ferroptosis bioprocesses, and through further validation at the in vitro cellular level and establishment of in vivo mouse tumor models, we have identified that TNBC abnormally high expression of the *IDH2* gene affected the degree of ferroptosis of TNBC cells by regulating the expression level of the *GPX4* gene, which in turn modulates the TNBC malignant biosignature.

## Discussion

Breast cancer has surpassed lung cancer as the most prevalent malignancy in humans, bringing the issues associated with the treatment of breast cancer back to the attention of a large number of researchers^1^. Although the widespread use of immune checkpoint inhibitors has brought partial hope for the treatment of TNBC, there is still no significant progress in the treatment of the vast majority of TNBC cases^5–7^. The discovery of the biological process of ferroptosis has provided novel ideas for the study of the mechanism of tumorigenesis and development^8–10^. To date, research on ferroptosis regulatory mechanisms has made some breakthroughs; not only has the classical *GPX4*-dependent pathway been evidenced in a variety of models, but other non-*GPX4*-dependent pathways have also gradually demonstrated crucial ferroptosis regulatory roles in different disease models^11–13^. Two biological enzymes, acyl coenzyme a synthase long-chain family member 4 (*ACSL4*) and lysophosphatidylcholine acyltransferase 3 (*LPCAT3*), serve as key enzymes in ferroptosis, and the role of *ACSL4* in ferroptosis is based on its predisposition to ligating long-chain PUFAs to coenzyme A, including arachidonic acid and adrenergic acid, which can then be passed through various LPCAT enzymes in the re-esterification of phospholipids^28,29^. The mutant condition of *ACSL4* deficiency allows *Gpx4* knockout cells to remain proliferative for months^29^, suggesting that the mechanism by which *GPX4* regulates ferroptosis still needs to be elucidated in depth. Notably, some studies have pointed out that the expression of *ACSL4* in TNBC cell lines correlates with their sensitivity to ferroptosis inducers; however, the diverse regulatory pathways of *ACSL4* are currently unclear^28,29^. Therefore, some scholars have suggested that the mechanism regulating ferroptosis may involve upstream regulators. Recently studies have gradually revealed that the biological pathway of ferroptosis has a critical role in the immunotherapy combination therapy of tumors, where CD8^+^ T cell activity is activated during tumor immunotherapy, whereas CD8^+^ T cells down-regulate the expression of the two subunits of the glutamate-cystine antagonist system xc-, SLC3A2 and SLC7A11, through the release of interferon-γ, which promotes tumor cell lipid peroxidation and ferroptosis^30^. Other studies have suggested that ferroptosis bioprocess inducers can enhance T cell and macrophage activity within the tumor immune microenvironment and have a synergistic effect in combination with immunotherapy, the exact mechanism of which is unclear and is currently thought to be related to the regulation of interferon^31,32^. Our results suggest that inhibition or activation of ferroptosis process has a significant role in the regulation of tumor proliferative capacity and in vivo tumourigenic capacity, but the effect did not meet therapeutic expectations, so we believe that the regulation of ferroptosis pathway is a key adjuvant measure in tumor therapy, and the correlation with the immune microenvironment or metabolic microenvironment needs to be further investigated.

**Figure 6:**
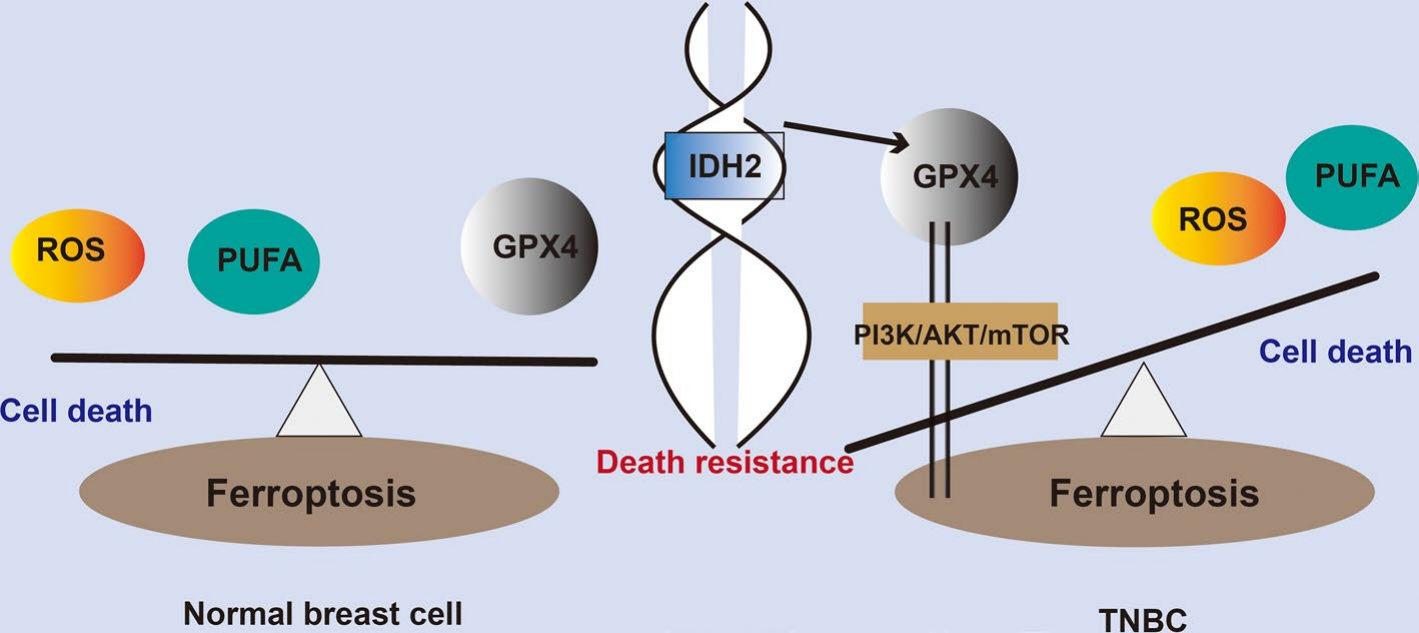
Schematic representation of the mechanism by which *IDH2* regulates the regulate cell death programme of TNBC cancer cells through ferroptosis bioprocesses.

Mutations in *IDH2*, a highly conserved gene in humans and other species, have been found to exist in a variety of disease models^23–25^, and its mutant metabolite D2-GH has been shown to have significant oncogenic effects^24^. Although a large number of studies have focused on the functional study of *IDH2* mutability, through large-throughput genomic analyses, scholars have continued to find that aberrant *IDH2* expression levels are more closely associated with disease development, with the most prominent function being related to energy metabolism^21,25,26^. In this study (Fig. 6), we first combined the abnormal expression level of *IDH2* with the regulation of ferroptosis, which not only verified that the unanticipated ferroptosis sensitivity shown by TCGA data analysis yielded the expected results in an in vitro model but also further suggested that *IDH2* might be a key gene upstream of the regulation of multiple ferroptosis pathways in TNBC. Based on the bioinformatics data, TCGA data and the recent studies, we can clearly recognise that there is a large variability in the biological process of ferroptosis in different breast cancer molecular subtypes, and even some key regulatory genes, such as *GPX4* and *IDH2*, have completely different expression statuses in different subtypes, and show opposite features in the clinical prognosis. Even in the TNBC subtype there is an extremely high degree of heterogeneity, which is not only evident in clinical samples but also in human breast cancer cell lines. As shown in Fig. 6, this study focuses on the biological process of ferroptosis in TNBC and screens for the specific regulatory gene *IDH2*, which is preliminarily demonstrated to significantly regulate ferroptosis-related genes and downstream pathways, and thus affect the proliferation ability of TNBC cancer cells themselves. For example, shRNA-*IDH2* not only significantly regulated the expression level of *GPX4* but also affected multiple ferroptosis-related genes, such as *ACSL4* and *COX1*, and we also found corresponding changes in the total amount and phosphorylation level of PI3K/AKT/mTOR downstream of multiple pathways, including ferroptosis, at the protein level.

Based on the data from a large number of other studies and the present study, we innovatively propose that *IDH2* has the potential to regulate multiple ferroptosis pathways upstream in TNBC, which in turn regulates the malignant characteristics of TNBC, such as high proliferative and migratory forces, and provides a new target and theoretical basis for the development of further clinical intervention strategies.

### Supplementary Information

Supplementary Figure 1.

Supplementary Figure 2.

Supplementary Table 1.

Supplementary Legends.

Supplementary Information 1.

Supplementary Information 2.

## Data Availability

The datasets presented in this study can be found in online repositories. The datasets generated and/or analysed during the current study are available in the online repository, https://www.ncbi.nlm.nih.gov/geo/ and http://www.sangerbox.com/tool. Dr. Yalong Yang should be contacted for other study data.

## Abbreviations

ACSL4: Acyl coenzyme a synthase long-chain family member 4
BRCA: Breast cancer
D2-HG: D-2-hydroxyglutaric acid
DEGs: Differentially expressed gene
ER: Estrogen receptor
FC: Flow cytometry
GFP: Green fluorescent protein
GPX4: Glutathione peroxidase 4
Her2: Human epidermal growth factor receptor 2
IDH2: Isocitrate dehydrogenase 2
IHC: Immunohistochemistry
LPCAT3: Lysophosphatidylcholine acyltransferase 3
PR: Progesterone receptor
PCA: Principal component analysis
PD-1: Programmed cell death protein 1
PDL-1: PD-1 ligand 1
PPI: Protein‒protein interaction network
TCGA: The Cancer Genome Atlas
TNBC: Triple-negative breast cancer
WB: Western blotting
WGCNA: Weighted gene coexpression network analysis
