# Supplementary figures and images for: Isocitrate dehydrogenase 2 regulates the proliferation of triple-negative breast cancer through the ferroptosis pathway
Source: Sci Rep. 2024 Feb 27;14:4732. doi: 10.1038/s41598-024-55561-0 (PMC10899212; doi:10.1038/s41598-024-55561-0)

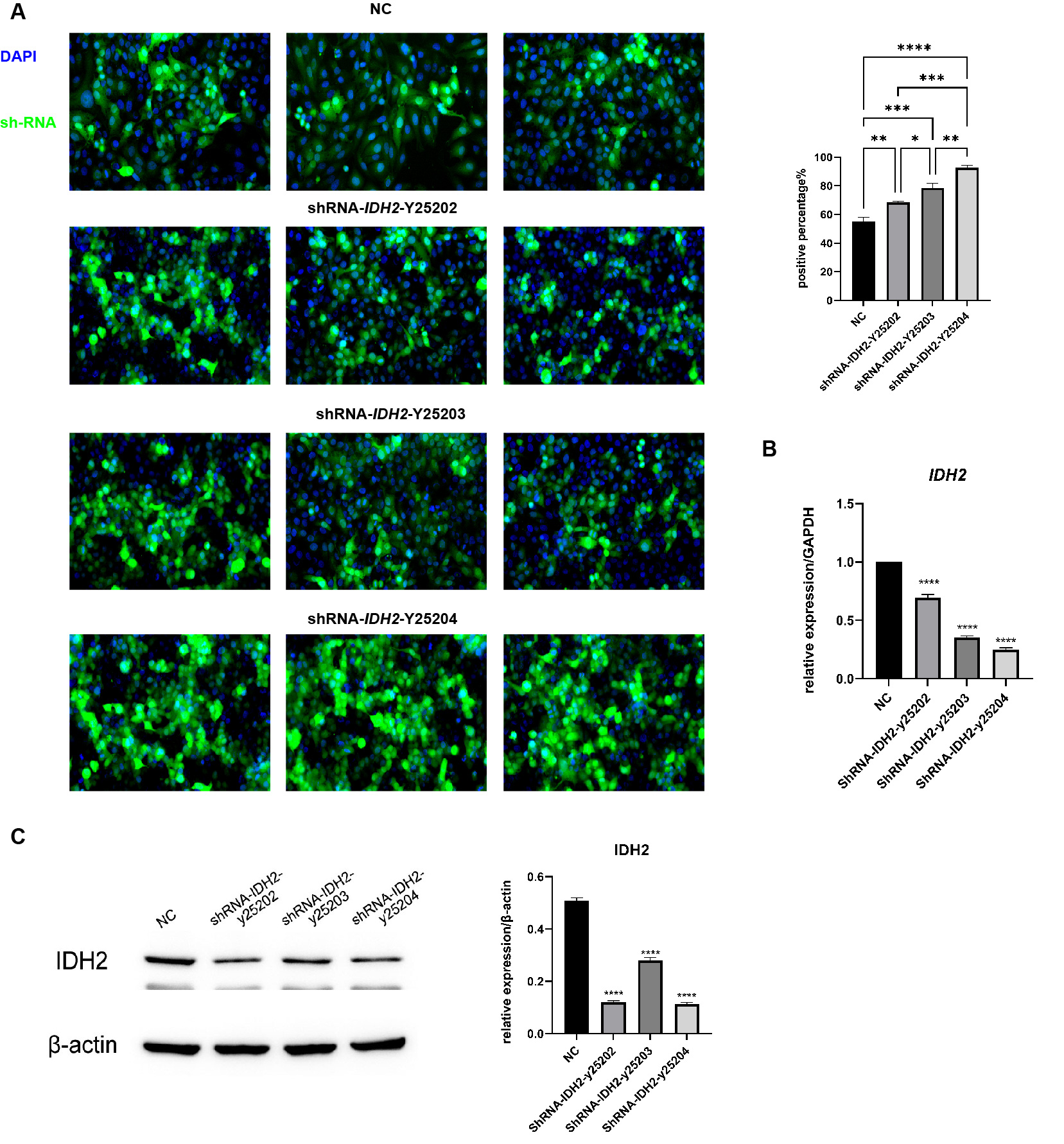

Supplement: Supplementary file 1 — Supplementary Figure 1. [file 41598_2024_55561_MOESM1_ESM.jpg]

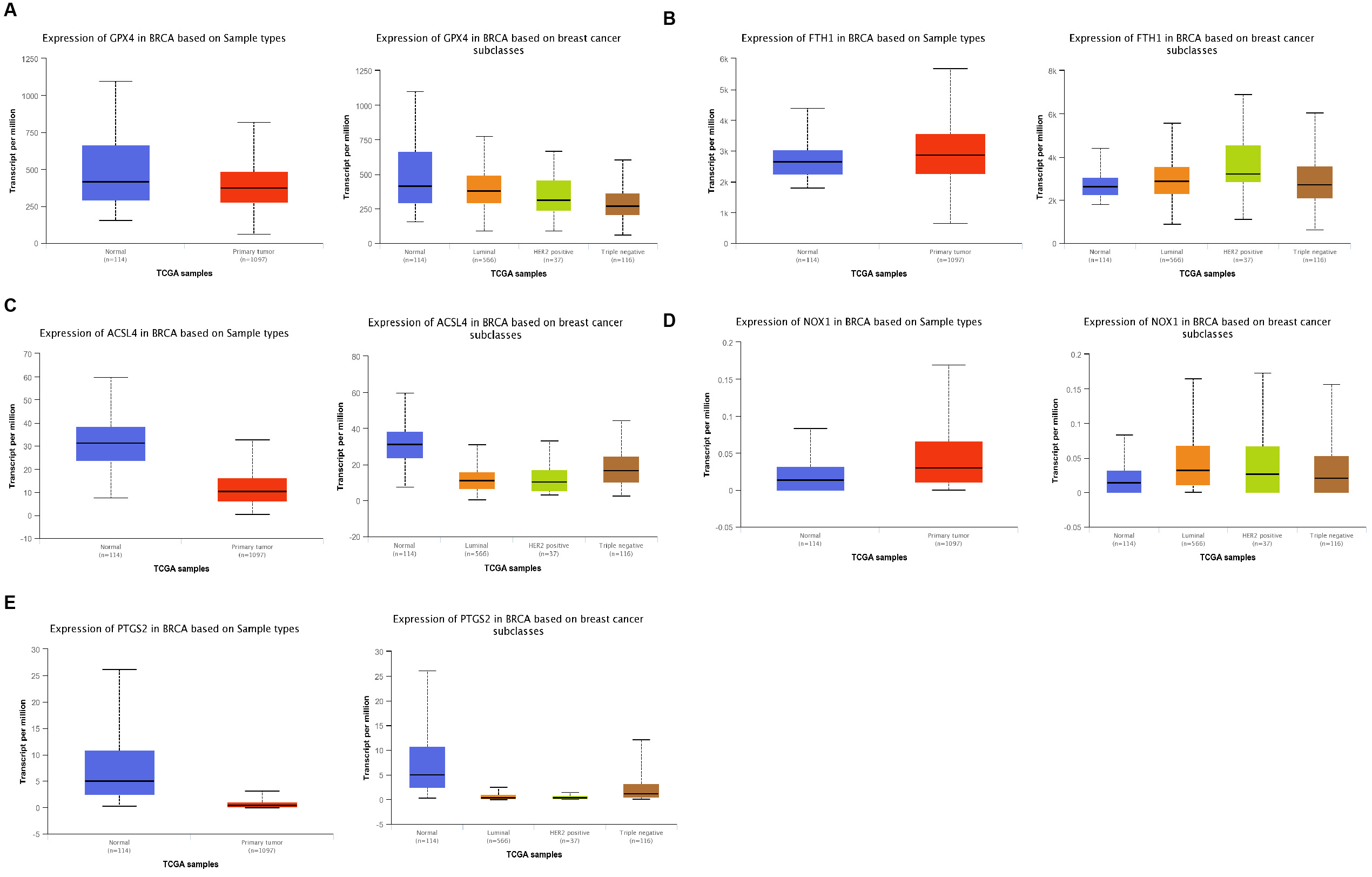

Supplement: Supplementary file 2 — Supplementary Figure 2. [file 41598_2024_55561_MOESM2_ESM.jpg]

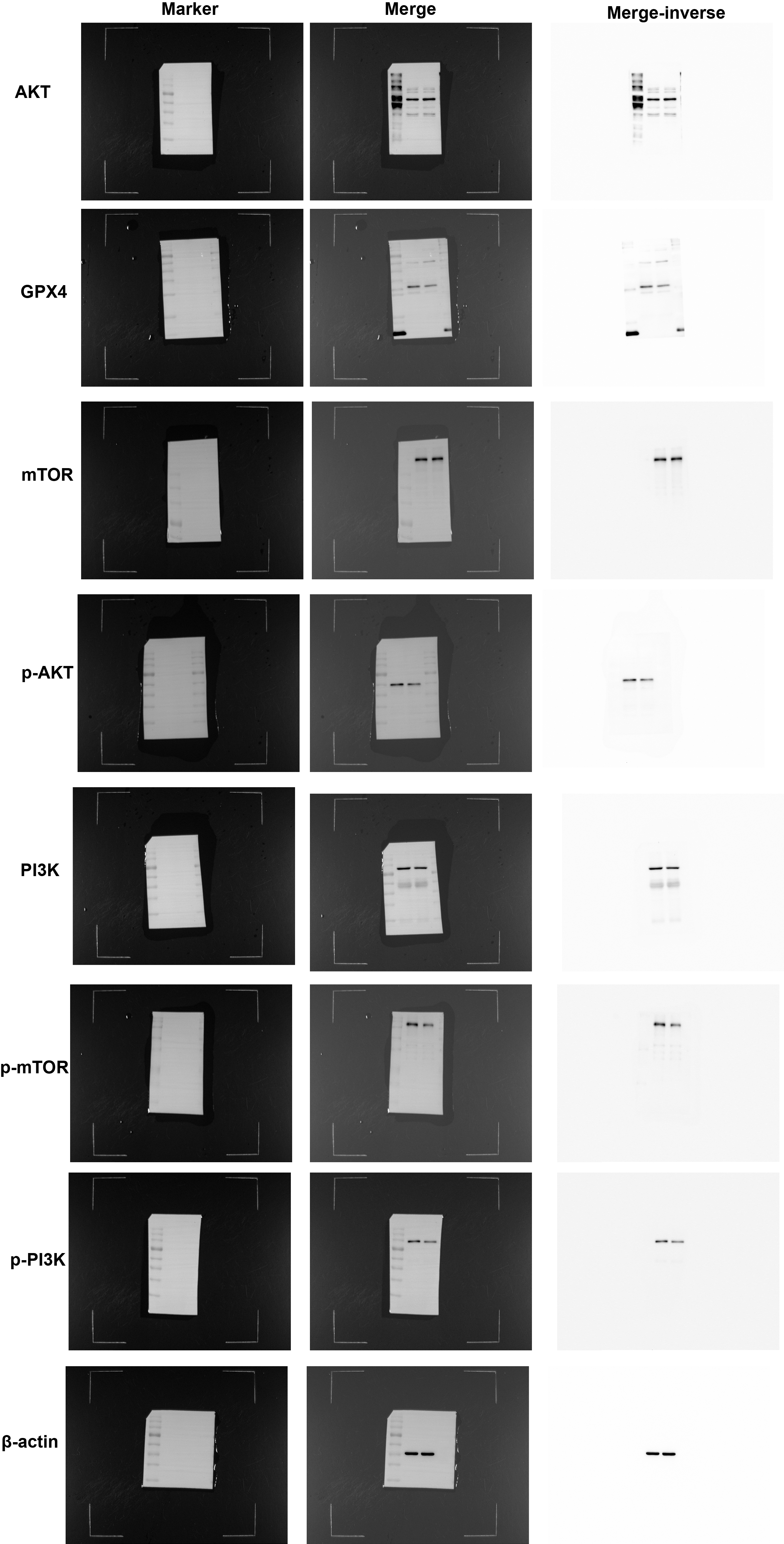

Supplement: Supplementary file 5 — Supplementary Information 1. [file 41598_2024_55561_MOESM5_ESM.jpg]

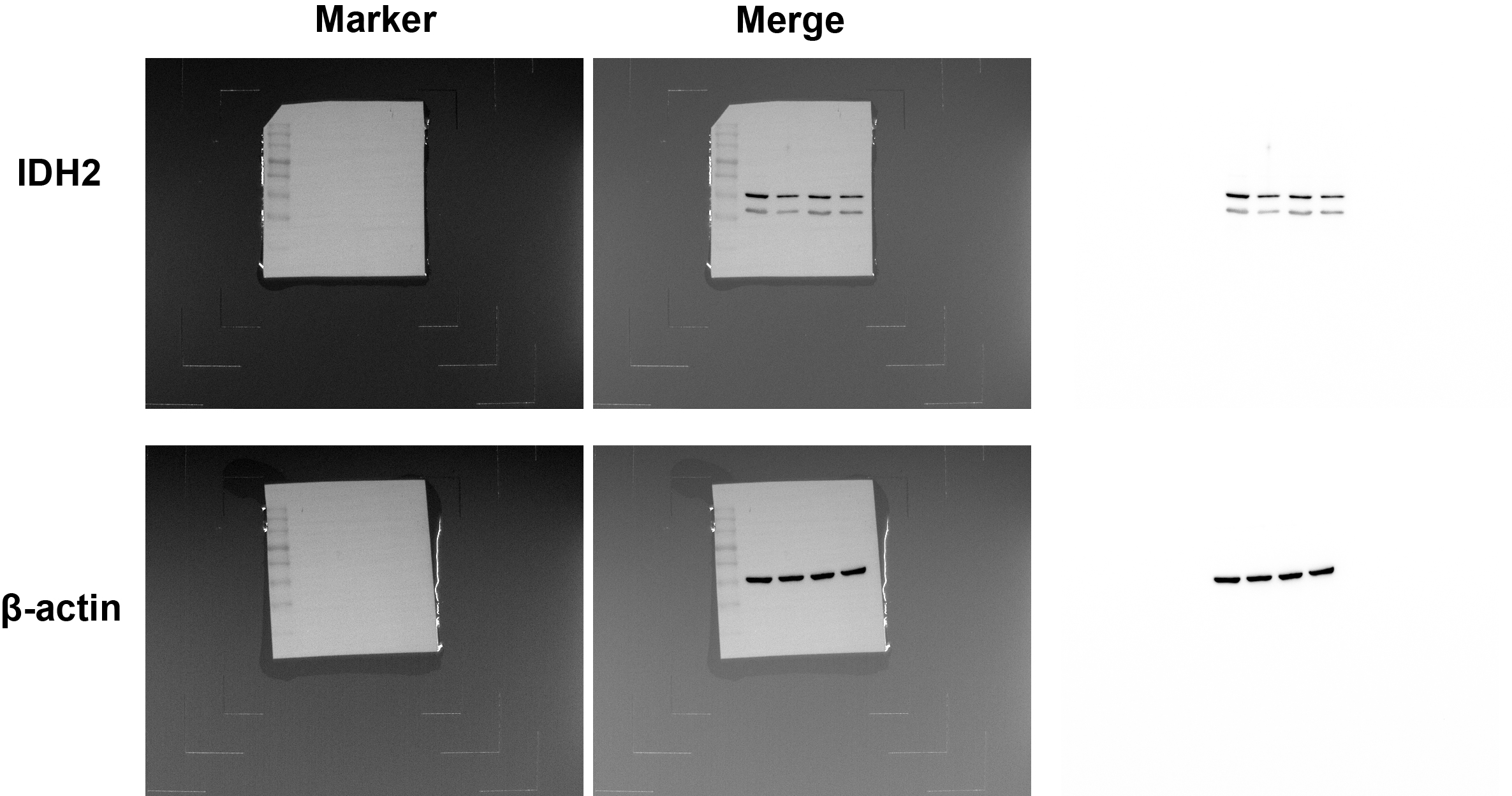

Supplement: Supplementary file 6 — Supplementary Information 2. [file 41598_2024_55561_MOESM6_ESM.jpg]
