# Supplementary material for: Isocitrate dehydrogenase 2 regulates the proliferation of triple-negative breast cancer through the ferroptosis pathway
Source: Sci Rep. 2024 Feb 27;14:4732. doi: 10.1038/s41598-024-55561-0 (PMC10899212; doi:10.1038/s41598-024-55561-0)
Supplement: Supplementary file 4 — Supplementary Legends. [file 41598_2024_55561_MOESM4_ESM.docx]

**SFigure 1. *IDH2* knockdown effect in MB-231 cells. A)** Three different groups of lentivirally transfected plasmids infected MB-231 cells, and the percentage of transfected nuclei with DAPI staining and GFP-labeled plasmids were observed under a fluorescence microscope after 24 h. Pictures were taken at 20x. **B)** The expression level of *IDH2* was measured by qRT‒PCR among different groups. n=4. **C)** IDH2 protein was collected from three different groups of lentivirally transfected plasmid-infected MDA-MB-231 cells at 48 h and was measured by WB compared with β-actin. Data were analyzed by one-way ANOVA followed by Tukey’s test. n=4. *, p <0.05, **, p <0.01, ***, p <0.005, ****, p < 0.001.

**SFigure 2. The expression of ferroptosis-related genes was analyzed through the TCGA database.** Data were analyzed via the Breast Cancer Gene-Expression Miner v5.0 (bc-GenExMiner v5.0) online tool. **A-B)** The anti-ferroptosis genes *GPX4* and *FTH1* and **C-E)** pro-ferroptosis genes *ACSL4, NOX1* and *PTGS2* were compared between normal breast samples with BRAC, as well as among different molecular types.

**STable 1. The sequences of different primers used in this study.**
